# Insomnia, Pre-Sleep Arousal, Psychosocial Factors and Changes in Sleep Pattern during the Second Wave Lockdown of the COVID-19 Pandemic in Georgia

**DOI:** 10.3390/brainsci12010017

**Published:** 2021-12-24

**Authors:** Tamar Basishvili, Nikoloz Oniani, Irine Sakhelashvili, Marine Eliozishvili, Manana Khizanashvili, Mariam Arabidze, Mariam Tsaava, Tinatini Charekishvili, Nino Tsertsvadze, Nato Darchia

**Affiliations:** Tengiz Oniani Laboratory of Sleep-Wakefulness Cycle Study, Ilia State University, Tbilisi 0162, Georgia; tamari.basishvili@iliauni.edu.ge (T.B.); nikoloz.oniani@iliauni.edu.ge (N.O.); irine.sakhelashvili.1@iliauni.edu.ge (I.S.); marine.eliozishvili@iliauni.edu.ge (M.E.); manana.khizanashvili.1@iliauni.edu.ge (M.K.); mariam.arabidze.1@iliauni.edu.ge (M.A.); mariam.tsaava.1@iliauni.edu.ge (M.T.); tinatini.charekishvili.1@iliauni.edu.ge (T.C.); nino.tsertsvadze.3@iliauni.edu.ge (N.T.)

**Keywords:** insomnia, somatic pre-sleep arousal, cognitive pre-sleep arousal, mental problems, COVID-19 pandemic waves

## Abstract

Studies performed across the COVID-19 pandemic waves point to the persistent impact of the pandemic on sleep and mental health. We expand these data by examining insomnia, pre-sleep arousal, psychosocial factors, and retrospective changes in sleep pattern during the COVID-19 second wave lockdown period in Georgia. Data were collected through an online survey (*n* = 1117). The prevalence rate of probable insomnia disorder was 24.2%. Clinically relevant somatic and cognitive pre-sleep arousal was present in 49.8% and 58.0% of participants, and high levels of anxiety, depression and social isolation were found in 47.0%, 37.3%, 47.2% of respondents, respectively. We observed high prevalence rates of worse sleep quality, delayed bedtimes and risetimes, longer sleep latencies, higher awakenings and shorter sleep durations, relative to the pre-pandemic period. COVID-19-infected participants showed more severe sleep and mental problems. Specific predictors differentially affected insomnia, somatic and cognitive pre-sleep arousal. Depression and COVID-19 infection emerged as vulnerability factors for pre-sleep arousal, which, in turn, was associated with a higher predisposition to insomnia disorder. We confirm the strong deteriorating impact of the COVID-19 pandemic on sleep and psychosocial well-being during the second wave lockdown period. The specific association between pre-sleep arousal, insomnia, and psychosocial factors is of clinical relevance for the prevention of severity and persistence of sleep and mental problems across the repeated lockdown/reopening waves. Modulation of pre-sleep arousal may prove beneficial to implement targeted interventions.

## 1. Introduction

The COVID-19 pandemic has encompassed every country and affected millions of people worldwide. The unprecedented social, psychological and economic problems that arose due to lockdown measures inflicted a prolonged stress on people, affected physical and mental health, and adversely impacted sleep-wake patterns and prevalence rates of sleep disorders [1,2,3]. Evidence indicates that sleep difficulties, among others, are the most frequent complaints during the COVID-19 pandemic period [2,3,4]. It has further been suggested that managing sleep problems during confinement may have a considerable benefit for mental health [5].

The measures undertaken to prevent the spread of COVID-19 vary across countries. The first COVID-19 confirmed case was detected in Georgia on 26 February 2020. On 21 March 2020, a state of emergency was announced in the country. On 31 March 2020, total quarantine was declared, which lasted a relatively short time, however several restrictions were in force. On 23 May 2020, the state of emergency ended, and the curfew was abolished. The second wave of the COVID-19 pandemic started in autumn, 2020. The number of confirmed cases in November–December 2020 significantly surpassed the case numbers during the first wave. Starting from 28 November, nationwide two-month restrictions were put in place, including a curfew from 21:00 h to 05:00 h, remote learning in schools and universities, a switch to online shopping by retailers, etc. Lockdown measures started to ease very slowly from 1 February 2021, and the ban on municipal transportation was lifted for weekdays from 8 February 2021 [6].

Although several studies have investigated the acute impact of the COVID-19 pandemic on sleep and mental health, data from follow-up pandemic waves are limited. At the same time, several waves of the pandemic crisis and consequently the variability of restrictions to combat the spread of severe acute respiratory syndrome coronavirus 2 (SARS-CoV-2) may expose people to uncertainty about getting back to a normal life routine, and even more robust changes in stress levels. Indeed, data from the Swiss Corona Stress study demonstrated a significant increase in stress levels and depressive symptoms during the second pandemic wave [7]. The persistent impact of the COVID-19 pandemic on sleep and psychological disturbances has been shown for the second wave in Italy [8]. Worsening of sleep quality, increase in the proportion of poor sleepers and different profiles of sleep patterns across the pandemic waves have been reported in another study of an Italian sample [9]. Significant changes in sleep quality, duration, and pre-sleep arousal were not revealed right after the end of the lockdown in an Italian sample [10]. Furthermore, mixed effects of the pandemic on the specific sleep quality domains in lockdown and post-lockdown periods were observed [11]. Several waves of the COVID-19 pandemic and associated challenges are most likely to have a strong impact on sleep and psychological wellbeing. Therefore, the present study was undertaken to: (a) examine prevalence rates and the relationship between insomnia, pre-sleep arousal and psychosocial factors, and (b) assess retrospective changes in sleep pattern during the second wave COVID-19 lockdown in Georgia. We focused on the relationship between insomnia and pre-sleep arousal, a topic that has largely been overlooked in COVID-19 research. We expected high prevalence rates of insomnia and clinically relevant pre-sleep arousal, as well as the various profiles of sleep pattern changes during the second wave lockdown period. We also expected that pre-sleep arousal would be a sensitive indicator of the COVID-19 pandemic’s impact on sleep and psychosocial well-being.

## 2. Materials and Methods

### 2.1. Study Design and Participants

A cross-sectional online survey was conducted among the general population of Georgia, aged 18 years or older, during the COVID-19 second wave lockdown period, from 15 January to 8 February 2021. The survey was administered using a Google form hosted by Ilya State University, Georgia, and has been disseminated through Ilia State University mail list, and on social media (Facebook). In addition, a snowball sampling method was applied, and participants were asked to disseminate the questionnaire through their own social network profiles. The study has been designed in accordance with the Declaration of Helsinki and was authorized by Ilia State University Research Ethics Committee (#140-35, 14 January 2021). Participants provided online informed consent. Incomplete questionnaires or questionnaires with impossible values (e.g., sleep duration longer than time in bed) were excluded from the analyses. From a total sample of 1263 participants surveyed, 1117 valid profiles were analyzed.

### 2.2. Measures

The structured questionnaire consisted of 6 subsections. The first subsection collected socio-demographic data such as age, sex, marital status (categorized into 2 groups: married/cohabiting or single/divorced/widowed), education (high school, student, University degree), employment status (employed, unemployed), economic status (bad, average, good), and information on health status (chronic disease, COVID-19 infection). Among those being infected with COVID-19, 19.7% were polymerase chain reaction (PCR)-confirmed, and 12.4% were suspected cases based on symptoms and a close contact with a diagnosed case, mostly family members. Since there were no significant differences when it comes to the main variables between confirmed and suspected COVID-19 cases, they were categorized into a single COVID-19-infected group.

The subsequent 4 subsections used the following validated questionnaires:

The Insomnia Severity Index (ISI) is a 7-item questionnaire assessing the severity of insomnia during the past month [12]. Each item is rated using a 5-point Likert scale from 0 (none) to 4 (very severe). The total ISI score is interpreted as follows: absence of insomnia (0–7), sub-threshold (8–14), moderate (15–21), and severe insomnia (22–28). In this study, the Cronbach’s α of the ISI-7 components score was 0.87.

The Perceived Stress Scale-4 (PSS-4) is a brief version of the original PSS-14 instrument [13]. It is a 4-item questionnaire designed to assess the respondent’s perception of stress on a 5-point scale from 0 (never) to 4 (very often). The Cronbach’s α of the PSS-4 in this study was 0.79.

The Pre-Sleep Arousal Scale (PSAS) is a 16-item questionnaire that assesses the symptoms of cognitive and somatic arousal experienced at bedtime [14]. Items are rated on a 5-point Likert scale ranging from 1 (not at all) to 5 (extremely). The sum of scores of the first 8 items measures somatic pre-sleep arousal (PSAS-somatic), and the last 8 item scores-cognitive pre-sleep arousal (PSAS-cognitive). The clinically relevant cut-off scores reported for PSAS-somatic and PSAS-cognitive are ≥14 and ≥20, respectively [15,16]. In the present study, the Cronbach’s α for the somatic scale was 0.82, for the cognitive scale –0.91.

Pittsburgh Sleep Quality Index (PSQI) is a widely used questionnaire that assesses 7 domains of sleep quality [17]. From PSQI, we only present bedtime, risetime, and sleep duration data. The follow-up publication will address the PSQI data in detail. The Cronbach’s α was 0.80 in the current sample.

Georgian versions of study instruments (ISI, PSS, PSAS, PSQI) have been validated, and showed good psychometric properties in the Georgian population, with details provided elsewhere [18,19,20].

Finally, the last subsection asked respondents to rate, retrospectively, changes in sleep-wake pattern, sleep quality and psychosocial variables, according to their experience in the past month (the same reference period as for questionnaires) compared to the pre-pandemic period; mainly, the survey queried about the changes in bedtime and risetime (delayed, unchanged, advanced); sleep latency, sleep duration and number of awakenings during the night (increased, unchanged, decreased); sleep quality, access to medical services and family environment (worse, unchanged, better). Finally, based on the validated assessment tools (Patient Health Questionnaire-9—PHQ-9; Generalized Anxiety Disorder 7-item scale—GAD-7), we formulated single questions and asked to rate how depressed (feeling sad, depressed, hopeless, little interest or pleasure in doing things), anxious (feeling anxious, worrying too much about different things, trouble relaxing) and socially isolated respondents felt during the past month, each on a 5-point scale from very low (1) to very high (5).

### 2.3. Statistical Analysis

Demographic, sleep, health and psychosocial variables were described using counts and percentages for categorical variables and means and standard deviations (SD) for continuous variables. The normal distribution of the study variables was tested (skewness range: −0.349–1.059; kurtosis range: −1.218–1.005). Chi-square, independent-sample t-tests and analysis of variance (ANOVA) were used for comparisons. Correlations between sleep and other variables were tested with Spearman or Pearson correlation, as appropriate. For hierarchical logistic regression analysis, individuals with ISI scores ≥15 were identified as the probable cases of insomnia disorder [21]. Block 1 of the regression model included all studied variables as predictors of probable insomnia disorder, except PSAS-somatic and PSAS-cognitive. Block 2 tested PSAS-somatic and PSAS-cognitive, as additional predictors. Finally, multiple linear regression analyses were conducted to examine the relationship between the demographic, health and psychosocial variables with PSAS-somatic and PSAS-cognitive, separately. All categorical variables were dummy coded, and all regression models were checked for multicolinearity by a variance inflation factor (VIF), which was below 2.5 for all variables. A two-tailed significance level was set at 0.05. The statistical analyses were performed using the Statistical Package for Social Sciences (SPSS) version 22.0.

## 3. Results

### 3.1. Sample Characteristics

The mean age of study participants was 38.5 (SD = 13.3; range 18–70) years, and the majority of them were female (86.6%). Overall, 48.9% of surveyed individuals were married or co-habiting, and 51.1% were single, divorced or widowed. The majority of respondents had a university degree (80.7%), were employed (71.8%), and reported an average household economic status (61.1%). Chronic disease was reported by 17.5% of the studied sample, and 32.1% of the participants were COVID-19-infected. Demographic, sleep, health and psychosocial characteristics of study participants as well as differences between COVID-19 and non-COVID-19 groups are presented in Table 1.

### 3.2. Sleep Pattern

**Figure 1:**
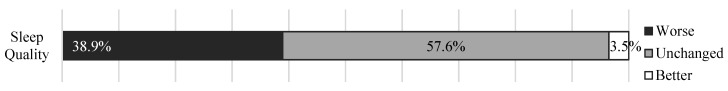

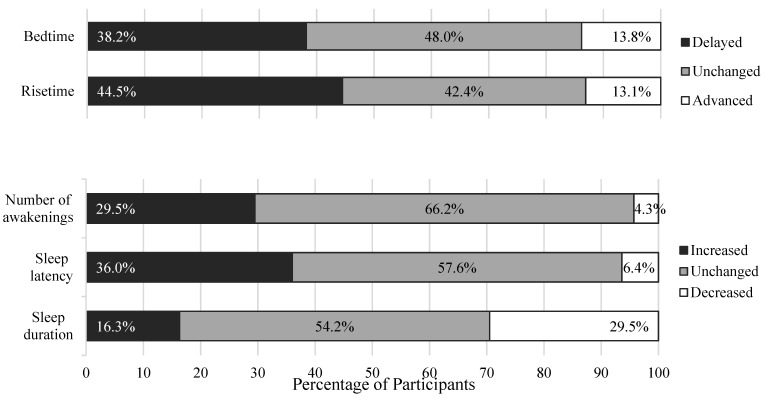
The retrospective changes in sleep pattern during the COVID-19 second wave lockdown period relative to the pre-pandemic time.

The mean bedtime for the whole sample was 00:50 ± 1:44 h. Of the entire sample, 31.8% went to bed before midnight, 46.9% between midnight and 02:00 h, and 21.3% went to bed at 02:00 h or later. A high number of participants (38.2%) reported going to bed later compared to the pre-pandemic period, while 13.8% reported an earlier bedtime. The mean sleep latency was 36.88 ± 35.62 min. Sleep latency longer than before the pandemic was revealed in 36.0% of the sample, while 6.4% reported a decrease in latency during the COVID-19 pandemic. The mean risetime of the studied population was 09:36 ± 1:58 h. Furthermore, 13.1% of participants reported an advanced and 44.5%-delayed risetime compared to the pre-pandemic period. The mean sleep duration was 7:06 ± 1:35 h. Sleep duration decreased in 29.5% and increased in 16.3% of the sample. Overall, 29.5% reported an increased number of awakenings during the night, and worse sleep quality was observed in 38.9% of the studied population. The retrospective comparison of the current sleep pattern to the pre-pandemic period is presented in Figure 1.

We also explored differences in sleep pattern between COVID-19-infected and non-infected groups. Infected participants went to bed earlier (*p* < 0.001) and had a longer sleep latency (*p* < 0.001). The difference in risetime was not significant (*p* = 0.93). There was a larger proportion of participants with a worse sleep quality (χ^2^ (2) = 29.177, *p* < 0.001), increased number of awakenings (χ^2^ (2) = 45.126, *p* < 0.001) and shorter sleep durations (χ^2^ (2) = 27.744, *p* < 0.001) in the COVID-19-infected group.

### 3.3. Insomnia and Pre-Sleep Arousal

Of the entire sample, 40.6% had sub threshold insomnia (ISI = 10.93 ± 2.03; mean ± SD) and 24.2% had moderate (ISI = 17.39 ± 1.97) to severe insomnia (ISI = 23.58 ± 1.37). Only 35.2% of respondents reported no insomnia symptoms with a mean ISI score of 3.91 ± 2.24. COVID-19-infected participants scored higher on the ISI compared to non-infected participants with a mean score of 11.17 ± 6.14 versus 9.78 ± 5.79, *p* < 0.001 (Table 1).

The ISI score showed the strongest positive correlations with PSAS-cognitive (r_s_ = 0.61, *p* < 0.001) and PSAS-somatic (r_s_ = 0.52, *p* < 0.001) scores. PSAS-somatic score increased significantly across four insomnia severity groups (no insomnia to severe insomnia) from 11.72 to 19.71 (F_(3,1113)_ = 116.762, *p* < 0.001). Likewise, compared to participants with no insomnia, those with a severe insomnia exhibited significantly higher PSAS-cognitive score (increase from 17.83 to 31.82, F_(3,1113)_ = 184.537, *p* < 0.001). The prevalence of individuals with clinically relevant pre-sleep arousal was substantial. In the total sample, 49.8% had PSAS-somatic and 58.0% had PSAS-cognitive above the cut-off scores. Furthermore, the proportion of participants with PSAS-somatic and PSAS-cognitive above the respective cut-off scores differed between the infected and non-infected individuals (58.8% vs. 45.5% for PSAS-somatic, χ^2^ (1) = 17.134, *p* < 0.001; 63.0% vs. 55.7% for PSAS-cognitive, χ^2^ (1) = 5.300, *p* < 0.05). Moreover, mean scores for PSAS-somatic (*p* < 0.001) and PSAS-cognitive (*p* < 0.01) were significantly higher in COVID-19-infected participants (Table 1).

### 3.4. Psychosocial Variables

Overall, a large proportion of respondents reported the high level of anxiety (47.0%), depression (37.3%) and social isolation (47.2%). Furthermore, 33.9% of respondents reported having more limited access to medical services compared to pre-pandemic period, and 45.6% reported worsening of the family environment. Comparing those outcomes between COVID-19 and non-COVID-19 participants, we found that there was a statistically significant difference with virus infected participants reporting a higher level of depression, anxiety, and worsened family environment (*p* < 0.05 for all). Similarly, PSS-4 mean score was significantly higher in COVID-19-infected participants (*p* < 0.05). Groups did not differ on social isolation and access to medical services (Table 1).

### 3.5. Predictors of Insomnia and Pre-Sleep Arousal

Table 2 presents results from hierarchical multiple logistic regression analyses aiming to identify the potential risk factors of insomnia disorder. The Block 1 model included socio-demographic, health and psychosocial variables. Model 2 tested PSAS-somatic and PSAS-cognitive, in addition to variables in Block 1. Both models were statistically significant (*p* < 0.001 for both). The Hosmer–Lemeshow Goodness test demonstrated a good fit for both models (*p* = 0.818, Block 1; *p* = 0.082, Block 2). Predictors of having probable cases of insomnia disorder (ISI scores ≥ 15) were COVID-19 infection, perceived stress, depression, and social isolation. Low education level approached the significance (*p* = 0.057). Adding PSAS-somatic and PSAS-cognitive variables to the model in Block 2 showed that COVID-19 infection and depression were no longer significant, while age and education exhibited a significant relationship (*p* < 0.05 for both), along with PSAS-somatic and PSAS-cognitive, both showing significant predictive capacity with sleep quality (*p* < 0.001 for both). PSAS-somatic and PSAS-cognitive, as predictors, increased the model’s predictive power from roughly 27.0% to 41.0% (Nagelkerke R^2^), and correctly classified 81.9% of the cases.

Correlation data between all the study variables are presented as the Supplementary Table S1.

Next, we tested the association of the same predictor variables with PSAS-somatic and PSAS-cognitive in a linear regression. The model with PSAS-somatic was significant (F_(18,1098)_ = 24.824, *p* < 0.001). Education (*p* < 0.05, high school; *p* < 0.01, being a student), chronic disease (*p* < 0.05), COVID-19 infection (*p* < 0.001), worse access to medical services (*p* < 0.05), stress appraisal (*p* < 0.001), anxiety (*p* < 0.001), and depression (*p* < 0.001) showed significant associations (Table 3). The model with PSAS-cognitive was also significant (F_(18,1098)_ = 35.578, *p* < 0.001) with following significant predictors—age (*p* < 0.001), being a student (*p* < 0.05), COVID-19 infection (*p* < 0.05), perceived stress (*p* < 0.001), anxiety (*p* < 0.001), depression (*p* < 0.001), and social isolation (*p* < 0.05). Association with employment status showed a trend toward significance (*p* = 0.07). The most significant predictor for both PSAS-somatic and PSAS-cognitive was perceived stress (Table 3).

## 4. Discussion

Scientific evidence that sleep problems severely affect people during the COVID-19 pandemic has rapidly emerged. To the best of our knowledge, this is a first study to explore sleep problems during the COVID-19 pandemic among the Georgian population, and one of the few studies assessing sleep and psychosocial problems during the second wave lockdown. Furthermore, this is a first study to examine the relationship between insomnia, PSAS-somatic, PSAS-cognitive and psychosocial factors during the COVID-19 pandemic period. Overall, our results confirm the strong impact of the pandemic on sleep and mental health. Our results show: (a) high prevalence rates of insomnia, clinically significant pre-sleep arousal and psychosocial problems; (b) higher severity of those problems in COVID-19-infected participants; (c) substantial changes in sleep pattern relative to the pre-pandemic time, retrospectively; (d) specific association between psychosocial factors, pre-sleep arousal, and insomnia. Mainly, in addition to common predictors, anxiety was a specific predictor of pre-sleep arousal; chronic disease and access to medical services predicted the PSAS somatic; social isolation was associated with PSAS cognitive and insomnia; depression and COVID-19 infection more likely affected insomnia through the impact on pre-sleep arousal.

The prevalence rate of probable cases of insomnia disorder in our study was 24.2% of the participants. This is comparable with the results of many previous surveys conducted in different countries during the first wave of the COVID-19 pandemic. In a study conducted in 13 countries during the first wave of the pandemic with a similar questionnaire and definition criteria for insomnia, the rate of probable clinical insomnia cases was 17.4% [3]. The pooled prevalence of insomnia in a meta-analysis of 22 studies was 48.0% [4]. There was a 26.7% increase in insomnia rates during the COVID-19 pandemic compared to the pre-pandemic data [22]. We observed about a two times higher rate of insomnia disorder in the present study compared with the rate reported in non-pandemic time in the Georgian population [19]. Although such comparisons are tentative, our findings indicate higher prevalence rates of sleep problems during the COVID-19 outbreak.

Psychosocial characteristics of study participants were also adversely affected by the pandemic. The proportion of respondents who reported high levels of feeling depressed (37.3%), anxious (47.0%) and socially isolated (47.2%) was substantial. The prevalence rates of depression and anxiety are comparable with mental health problems reported in the Georgian population during the pandemic period [23], as well as being consistent with other COVID-19 studies [3,24,25], although we note the obvious differences in methodologies.

Our data furthermore corroborate findings of studies addressing sleep and mental health in different COVID-19 pandemic waves. Salfi et al. [8] reported a persistent impact of the pandemic on sleep and mental disturbances across the first and second waves. Another study reported that sleep quality returned to the baseline level after the first lockdown and then worsened again during the second lockdown period [9]. In contrast, the prevalence of insomnia, depression and anxiety did not differ between the COVID-19 lockdown period and 6 months after the start of lockdown in an Austrian sample [26]. The possible role of stress in modulating sleep changes across the pandemic waves has been proposed [11]. These data and our own results indicate that the negative impact of the pandemic on sleep and mental wellbeing persists over time.

In line with other studies, we demonstrated the strong relationship between insomnia and psychosocial variables. In addition, we provide evidence on some aspects of this relationship not yet addressed, such as the role of the pre-sleep arousal. Although pre-sleep arousal plays an important role in the relationship between stress and sleep [27], we found only two studies examining pre-sleep arousal during the COVID-19 pandemic. One study evaluated the effect of cognitive behavioral therapy for insomnia (CBT-I) intervention for situational insomnia [28]. The other study comprehensively assessed the relationship of PSAS-somatic and PSAS-cognitive with sleep quality [16]. The number of individuals reporting clinically relevant levels of PSAS-somatic and PSAS-cognitive (49.8% and 58%) in our study were somewhat higher than those reported by Gorgoni et al. [16] for the first lockdown period, which may account for the difference in results. These findings further suggest that the impact of the COVID-19 pandemic persists and may even worsen across the pandemic waves. Beyond confirming high rates of clinically relevant pre-sleep arousal during the COVID-19 pandemic reported by Gorgoni et al. [16], we also showed that various factors differentially affected insomnia disorder, PSAS-somatic and PSAS-cognitive. First, we observed that COVID-19 infection, education, stress, depression, social isolation, but not anxiety, predicted clinical insomnia. It is plausible that during the second wave of the pandemic, people experienced less anxiety in adjusting their lifestyle routines to the confinement. Salfi et al.’s [8] findings of a reduced severity of anxiety symptoms but a stable severity of depressive symptoms between two pandemic waves support this interpretation. We also demonstrated that PSAS-somatic and PSAS-cognitive were strongly associated with insomnia (14.3% increase in R^2^), but depression and COVID-19 infection failed to remain significant in this association. At the same time, both depression and COVID-19 infection were associated with PSAS-somatic and PSAS-cognitive. Therefore, it seems more likely that depression and COVID-19 infection were vulnerability factors for pre-sleep arousal, which, in turn, was associated with a higher predisposition to insomnia disorder. On the other hand, perceived stress predicted both insomnia severity and pre-sleep arousal level, similar to Gorgoni et al.’s [16] findings for sleep quality. Given that personal vulnerability to stressful events plays a role in developing insomnia [27,29], these results are not surprising. Our results are consistent with the role of perceived stress during the COVID-19 lockdown in developing sleep problems [30]. Although the decrease of perceived stress 6 months after the COVID-19 lockdown has also been reported, the effect was not clinically relevant [26]. The novelty of stressor and uncertainty with the pandemic-associated consequences could have had a greater impact on stress appraisal during the first lockdowns. However, our results suggest that the appraisal of stressors, and thus personal vulnerability to stressful events, is a long-lasting operative factor in the COVID-19 pandemic’s impact on health. Finally, we agree with Gorgoni et al.’s [16] suggestion that the evaluation of pre-sleep arousal and associated factors during the COVID-19 pandemic can be beneficial in preventing an increase in insomnia prevalence.

We observed a greater predisposition of socially isolated individuals to insomnia, consistent with the literature [31]. The positive impact of social support on sleep quality is well known [32]. Our data showed that feeling socially isolated represents a predictor of insomnia and PSAS-cognitive, further highlighting its role in developing sleep problems. This fact correlates with the finding that individuals living alone are at higher risk for developing insomnia [3]. Therefore, the beneficial role of social interaction to reduce the burden of sleep problems during the pandemic should be encouraged (e.g., social interaction with family members; reasonable use of technologies for social connection with friends despite the need for social distancing).

Crucially, we revealed that COVID-19-infected participants were more likely to experience insomnia and have a higher level of anxiety, depression, perceived stress, PSAS-somatic and PSAS-cognitive. Higher risks of insomnia in those who had COVID-19 infection were also reported in a recent multi-center study [3]. The prevalence rate of insomnia in COVID-19 inpatients in China was very high (42.8%) and was associated with severity of anxiety [33]. Therefore, the sleep and mental health of COVID-19-infected individuals are of immediate clinical importance to avoid long-lasting sleep problems and psychopathologies.

Finally, the negative impact of the COVID-19 pandemic on sleep pattern, frequently reported in the literature [34,35,36], was also observed in the present study. A high number of participants reported delayed bed- and rise times (38.2% and 44.5%), longer sleep latencies (36.0%), a higher number of awakenings (29.5%) and shorter sleep durations (29.5%) compared to how they slept before the pandemic. As in other studies, there were a low proportion of participants who reported the advancement of sleep time variables, and 3.5% reported better sleep quality, supporting the possibility that weakened social constraints on sleep-wake schedule may have a beneficial effect in some individuals [37,38].

The present study has some limitations. First, the study was based on a cross-sectional design. Second, there might be a recruitment bias due to the online survey. In addition, the retrospective assessment of some variables could have led to the recall bias. Using single questions for the evaluation of depression and anxiety, and merging the PCR confirmed and suspected cases into a single group should be acknowledged. Finally, the higher proportion of females (86.6%) in this study, as in many online COVID-19 surveys, may have had an impact on prevalence rates of insomnia and clinically relevant pre-sleep arousal [39,40].

## 5. Conclusions

Our findings demonstrated the complex array of changes in sleep pattern, and alarming prevalence rates of insomnia and psychosocial problems, especially in those with COVID-19 infection. Therefore, increased attention towards distinct profiles of sleep and mental problems during and after repeated cycles of the lockdown and reopening is required, especially for those at risk. Furthermore, given the specific association between pre-sleep arousal, insomnia and psychosocial factors, underlined in the present study, the modulation of pre-sleep arousal level (e.g., adaptations of CBT and mindfulness-based therapy elements; relaxation techniques) may prove more beneficial to counteract the alarming prevalence rates of clinically relevant insomnia in the pandemic crises, and to minimize long-lasting sleep and mental disturbances. Public sleep awareness and health prevention programs to address diverse profiles of sleep problems across the COVID-19 pandemic waves are necessary. Several approaches, such as tips for healthy sleep practices; CBT-I; mindfulness-based therapy for insomnia (MBTI); mindfulness-based stress reduction (MBSR), can be adapted and accessible via web-based platforms.

## Figures and Tables

**Table 1:** Demographic, health and psychosocial variables within the whole sample and separated by the COVID-19 infection.

	Total Sample *n* = 1117	COVID-19 *n* = 359	Non-COVID-19 *n* = 758	Statistics
Age	38.50 ± 13.30	37.37 ± 12.90	39.03 ± 13.40	t_(1115)_ = −1.954, *p* = 0.051
Sex				
Male Female	150 (13.4%) 967 (86.6%)	36 (10.0%) 323 (90.0%)	114 (15.0%) 644 (85.0%)	χ^2^ (1) = 5.263, *p* < 0.05
Marital status				
Married/cohabiting Single/divorced/widowed	546 (48.9%) 571 (51.1%)	183 (51.0%) 176 (49.0%)	363 (47.9%) 395 (52.1%)	χ^2^ (1) = 0.928, *p* = 0.335
Education				
University degree High school Student	902 (80.7%) 59 (5.3%) 156 (14.0%)	274 (76.3%) 30 (8.4%) 55 (15.3%)	628 (82.9%) 29 (3.8%) 101 (13.3%)	χ^2^ (2) = 11.447, *p* < 0.01
Employment				
Yes No	802 (71.8%) 315 (28.2%)	232 (64.6%) 127 (35.4%)	570 (75.2%) 188 (24.8%)	χ^2^ (1) = 13.453, *p* < 0.001
Economic status				
Good Average Bad	213 (19.1%) 683 (61.1%) 221 (19.8%)	50 (13.9%) 216 (60.2%) 93 (25.9%)	163 (21.5%) 467 (61.6%) 128 (16.9%)	χ^2^ (2) = 17.432, *p* < 0.001
Chronic disease				
Yes No	196 (17.5%) 921 (82.5%)	71 (19.8%) 288 (80.2%)	125 (16.5%) 633 (83.5%)	χ^2^ (1) = 1.819, *p* = 0.177
Access to medical services				
Worse No change Better	379 (33.9%) 718 (64.3%) 20 (1.8%)	126 (35.1%) 222 (61.8%) 11 (3.1%)	253 (33.4%) 496 (65.4%) 9 (1.2%)	χ^2^ (2) = 5.495, *p* = 0.064
Family environment				
Worse No change Better	509 (45.6%) 570 (51.0%) 38 (3.4%)	185 (51.5%) 166 (46.3%) 8 (2.2%)	324 (42.7%) 404 (53.3%) 30 (4.0%)	χ^2^ (2) = 8.649, *p* < 0.05
Anxiety	3.41 ± 1.17	3.53 ± 1.17	3.36 ± 1.17	t_(1115)_ = −2.238, *p* < 0.05
Depression	3.03 ± 1.33	3.14 ± 1.33	2.97 ± 1.32	t_(1115)_ = −1.984, *p* < 0.05
Social Isolation	3.37 ± 1.19	3.37 ± 1.15	3.37 ± 1.20	t_(1115)_ = 0.057, *p* = 0.954
ISI	10.23 ± 5.94	11.17 ± 6.14	9.78 ± 5.79	t_(1115)_ = 3.663, *p* < 0.001
PSS-4	6.80 ± 2.95	7.13 ± 2.94	6.64 ± 2.94	t_(1115)_ = 2.599, *p* < 0.01
PSAS-Somatic	14.62 ± 5.40	15.96 ± 5.89	13.99 ± 5.03	t_(1115)_ = 5.781, *p* < 0.001
PSAS-Cognitive	22.07 ± 7.28	23.13 ± 7.43	21.57 ± 7.16	t_(1115)_ = 3.348, *p* < 0.01

Data are presented as the means and standard deviations or counts and percentages. ISI, Insomnia severity index; PSS-4, perceived stress scale-4; PSAS-Somatic, Somatic pre-sleep arousal; PSAS-Cognitive, Cognitive pre-sleep arousal.

**Table 2:** Prediction of insomnia disorder (ISI ≥ 15) based on the logistic regression models (*n* = 1117).

Predictors	Model 1			Model 2		
	OR	95% CI	*p*	OR	95% CI	*p*
Age	1.01	0.99–1.02	0.481	1.02	1.00–1.04	0.033
Sex						
Female Male	Reference 1.22	0.75–1.98	0.421	1.37	0.82–2.31	0.232
Marital status						
Married/cohabiting Single/divorced/widowed	Reference 0.87	0.62–1.23	0.432	0.83	0.57–1.20	0.322
Education						
University High school Student	Reference 1.83 1.22	0.98–3.43 0.72–2.06	0.060 0.459	2.03 0.90	1.03–4.01 0.50–1.62	0.042 0.728
Employment						
Employed Unemployed	Reference 0.81	0.56–1.17	0.264	0.91	0.61–1.36	0.629
Economic status						
Good Average Bad	Reference 1.11 1.32	0.69–1.77 0.77–2.28	0.669 0.315	1.08 1.39	0.65–1.81 0.76–2.53	0.757 0.282
Chronic disease						
No Yes	Reference 1.28	0.85–1.93	0.229	1.20	0.77–1.87	0.419
COVID-19 infection						
No Yes	Reference 1.56	1.13–2.16	0.007	1.33	0.93–1.89	0.120
Access to medical services						
No change Worse Better	Reference 1.26 2.09	0.91–1.75 0.70–6.23	0.158 0.185	1.29 2.81	0.90–1.84 0.85–9.32	0.161 0.091
Family environment						
No change Worse Better	Reference 1.15 1.09	0.83–1.61 0.45–2.63	0.403 0.854	1.14 0.93	0.79–1.64 0.35–2.46	0.487 0.877
Anxiety	1.16	0.97–1.40	0.111	0.92	0.75–1.13	0.425
Depression	1.22	1.03–1.45	0.023	1.09	0.90–1.31	0.374
Social isolation	1.26	1.08–1.47	0.004	1.24	1.05–1.47	0.012
PSS-4	1.29	1.21–1.38	0.000	1.16	1.08–1.25	0.000
PSAS-Somatic				1.07	1.03–1.11	0.001
PSAS-Cognitive				1.15	1.11–1.18	0.000
Nagelkerke R^2^	0.269			0.412		
Correct classification (%)	78.5%			81.9%		

ISI, Insomnia severity index; OR, Odds ratio; CI, confidence interval; PSS-4, Perceived stress scale-4; PSAS-Somatic, Somatic pre-sleep arousal; PSAS-Cognitive, Cognitive pre-sleep arousal.

**Table 3:** Multiple linear regression results for PSAS-somatic and PSAS-cognitive.

Predictors	PSAS-Somatic		PSAS-Cognitive	
	β	*p*	β	*p*
Age	0.025	0.448	−0.126	0.000
Sex				
Female Male	Reference −0.001	0.968	−0.006	0.816
Marital status				
Married/cohabiting Single/divorced/widowed	Reference −0.002	0.951	0.018	0.509
Education				
University High school Student	Reference 0.057 0.096	0.031 0.003	−0.014 0.080	0.584 0.010
Employment				
Employed Unemployed	Reference −0.006	0.839	−0.049	0.070
Economic status				
Good Average Bad	Reference 0.008 0.013	0.801 0.703	0.006 −0.004	0.848 0.909
Chronic disease				
No Yes	Reference 0.066	0.013	0.007	0.788
COVID-19 infection				
No Yes	Reference 0.118	0.000	0.053	0.030
Family environment				
No change Worse Better	Reference 0.033 0.020	0.241 0.456	0.001 −0.001	0.978 0.960
Access to medical services				
No change Worse Better	Reference 0.066 0.000	0.014 0.999	0.005 −0.003	0.836 0.904
PSS-4	0.234	0.000	0.302	0.000
Anxiety	0.167	0.000	0.195	0.000
Depression	0.173	0.000	0.126	0.000
Social isolation	−0.021	0.475	0.071	0.010
Adjusted R^2^	28.9%		35.8%	

PSAS-Somatic, Somatic pre-sleep arousal; PSAS-Cognitive, Cognitive pre-sleep arousal; PSS-4, Perceived stress scale-4; β, Standardized regression coefficient.

## Data Availability

The data that support the findings of this study are available from the corresponding author upon reasonable request.

## Supplementary Materials

The following is available online at https://www.mdpi.com/article/10.3390/brainsci12010017/s1, Table S1: Pearson’s and Spearman’s correlations between all the study variables

## References

[1] Vinkers C.H., van Amelsvoort T., Bisson J.I., Branchi I., Cryan J.F., Domschke K., Howes O.D., Manchia M., Pinto L., de Quervain D. (2020). Stress resilience during the coronavirus pandemic. Eur. Neuropsychopharmacol..

[2] Jahrami H., BaHammam A.S., Bragazzi N.L., Saif Z., Faris M., Vitiello M.V. (2021). Sleep problems during the COVID-19 pandemic by population: A systematic review and meta-analysis. J. Clin. Sleep Med..

[3] Morin C.M., Bjorvatn B., Chung F., Holzinger B., Partinen M., Penzel T., Ivers H., Wing Y.K., Chan N.Y., Merikanto I. (2021). Insomnia, anxiety, and depression during the COVID-19 pandemic: An international collaborative study. Sleep Med..

[4] Liu C., Pan W., Li L., Li B., Ren Y., Ma X. (2021). Prevalence of depression, anxiety, and insomnia symptoms among patients with COVID-19: A meta-analysis of quality effects model. J. Psychosom. Res..

[5] Altena E., Baglioni C., Espie C.A., Ellis J., Gavriloff D., Holzinger B., Schlarb A., Frase L., Jernelöv S., Riemann D. (2020). Dealing with sleep problems during home confinement due to the COVID-19 outbreak: Practical recommendations from a task force of the European CBT-I Academy. J. Sleep Res..

[6] NCDC (2021). One year with COVID-19. Report of the National Center for Disease Control and Public Health.

[7] De Quervain D., Aerni A., Amini E., Bentz D., Coynel D., Gerhards C., Fehlmann B., Freytag V., Papassotiropoulos A., Schicktanz N. (2020). The Swiss Corona Stress Study. https://osf.io/jqw6a/.

[8] Salfi F., D’Atri A., Tempesta D., Ferrara M. (2021). Sleeping under the waves: A longitudinal study across the contagion peaks of the COVID-19 pandemic in Italy. J. Sleep Res..

[9] Conte F., Cellini N., de Rosa O., Rescott M.L., Malloggi S., Giganti F., Ficca G. (2021). Dissociated profiles of sleep timing and sleep quality changes across the first and second wave of the COVID-19 pandemic. J. Psychiatr. Res..

[10] Gorgoni M., Scarpelli S., Mangiaruga A., Alfonsi V., Bonsignore M.R., Fanfulla F., Ferini-Strambi L., Nobili L., Plazzi G., De Gennaro L. (2021). Persistence of the Effects of the COVID-19 Lockdown on Sleep: A Longitudinal Study. Brain Sci..

[11] Alfonsi V., Gorgoni M., Scarpelli S., Zivi P., Sdoia S., Mari E., Fraschetti A., Ferlazzo F., Giannini A.M., De Gennaro L. (2021). COVID-19 lockdown and poor sleep quality: Not the whole story. J. Sleep Res..

[12] Bastien C.H., Vallières A., Morin C.M. (2001). Validation of the Insomnia Severity Index as an outcome measure for insomnia research. Sleep Med..

[13] Cohen S., Williamson G., Spacapam S., Oskamp S. (1988). Perceived stress in a probability sample of the United States. The Social Psychology of Health.

[14] Nicassio P.M., Mendlowitz D.R., Fussell J.J., Petras L. (1985). The phenomenology of the pre-sleep state: The development of the pre-sleep arousal scale. Behav. Res. Ther..

[15] Puzino K., Amatrudo G., Sullivan A., Vgontzas A.N., Fernandez-Mendoza J. (2020). Clinical Significance and Cut-Off Scores for the Pre-Sleep Arousal Scale in Chronic Insomnia Disorder: A Replication in a Clinical Sample. Behav. Sleep Med..

[16] Gorgoni M., Scarpelli S., Mangiaruga A., Alfonsi V., Bonsignore M.R., Fanfulla F., Ferini-Strambi L., Nobili L., Plazzi G., De Gennaro L. (2021). Pre-sleep arousal and sleep quality during the COVID-19 lockdown in Italy. Sleep Med..

[17] Buysse D.J., Reynolds C.F., Monk T.H., Berman S.R., Kupfer D.J. (1989). The Pittsburgh Sleep Quality Index: A new instrument for psychiatric practice and research. Psychiatry Res..

[18] Sakhelashvili I., Eliozishvili M., Basishvili T., Datunashvili M., Oniani N., Cervena K., Darchia N. (2016). Sleep-wake patterns and sleep quality in urban Georgia. Transl. Neurosci..

[19] Darchia N., Oniani N., Sakhelashvili I., Supatashvili M., Basishvili T., Eliozishvili M., Maisuradze L., Cervena K. (2018). Relationship between Sleep Disorders and Health Related Quality of Life-Results from the Georgia SOMNUS Study. Int. J. Environ. Res. Public Health.

[20] Sakhelashvili I., Eliozishvili M., Oniani N., Darchia N., Bruni O. (2018). Sleep and psycho-behavioral problems in internally displaced children in Georgia. Sleep Med..

[21] Morin C.M., Belleville G., Bélanger L., Ivers H. (2011). The Insomnia Severity Index: Psychometric indicators to detect insomnia cases and evaluate treatment response. Sleep.

[22] Morin C.M., Vézina-Im L.A., Ivers H., Micoulaud-Franchi J.A., Philip P., Lamy M., Savard J. (2021). Prevalent, Incident, and Persistent Insomnia in a Population-Based Cohort Tested Before (2018) and During the First-Wave of COVID-19 Pandemic (2020). Sleep.

[23] Makhashvili N., Javakhishvili J.D., Sturua L., Pilauri K., Fuhr D.C., Roberts B. (2020). The influence of concern about COVID-19 on mental health in the Republic of Georgia: A cross-sectional study. Glob. Health.

[24] Casagrande M., Favieri F., Tambelli R. (2020). The enemy who sealed the world: Effects quarantine due to the COVID-19 on sleep quality, anxiety, and psychological distress in the Italian population. Sleep Med..

[25] Huang Y., Zhao N. (2020). Generalized anxiety disorder, depressive symptoms and sleep quality during COVID-19 outbreak in China: A web-based cross-sectional survey. Psychiatry Res..

[26] Pieh C., Budimir S., Humer E., Probst T. (2021). Comparing Mental Health during the COVID-19 Lockdown and 6 Months after the Lockdown in Austria: A Longitudinal Study. Front. Psychiatry.

[27] Morin C.M., Rodrigue S., Ivers H. (2003). Role of stress, arousal, and coping skills in primary insomnia. Psychosom. Med..

[28] Zhang C., Yang L., Liu S., Xu Y., Zheng H., Zhang B. (2021). One-Week Self-Guided Internet Cognitive Behavioral Treatments for Insomnia in Adults with Situational Insomnia During the COVID-19 Outbreak. Front. Neurosci..

[29] Basishvili T., Eliozishvili M., Maisuradze L., Lortkipanidze N., Nachkebia N., Oniani T., Gvilia I., Darchia N. (2012). Insomnia in a displaced population is related to war-associated remembered stress. Stress Health.

[30] Zhao X., Lan M., Li H., Yang J. (2021). Perceived stress and sleep quality among the non-diseased general public in China during the 2019 coronavirus disease: A moderated mediation model. Sleep Med..

[31] Xiao H., Zhang Y., Kong D., Li S., Yang N. (2020). The Effects of Social Support on Sleep Quality of Medical Staff Treating Patients with Coronavirus Disease 2019 (COVID-19) in January and February 2020 in China. Med. Sci. Monit..

[32] Kent de Grey R.G., Uchino B.N., Trettevik R., Cronan S., Hogan J.N. (2018). Social support and sleep: A meta-analysis. Health Psychol..

[33] Wang Y., Zhu L.Y., Ma Y.F., Bo H.X., Deng H.B., Cao J., Wang Y., Wang X.J., Xu Y., Lu Q.D. (2020). Association of insomnia disorder with sociodemographic factors and poor mental health in COVID-19 inpatients in China. Sleep Med..

[34] Beck F., Léger D., Fressard L., Peretti-Watel P., Verger P., Coconel Group (2021). COVID-19 health crisis and lockdown associated with high level of sleep complaints and hypnotic uptake at the population level. J. Sleep Res..

[35] Lin L.Y., Wang J., Ou-Yang X.Y., Miao Q., Chen R., Liang F.X., Zhang Y.P., Tang Q., Wang T. (2021). The immediate impact of the 2019 novel coronavirus (COVID-19) outbreak on subjective sleep status. Sleep Med..

[36] Robillard R., Dion K., Pennestri M.H., Solomonova E., Lee E., Saad M., Murkar A., Godbout R., Edwards J.D., Quilty L. (2021). Profiles of sleep changes during the COVID-19 pandemic: Demographic, behavioural and psychological factors. J. Sleep Res..

[37] Kocevska D., Blanken T.F., Van Someren E.J.W., Rösler L. (2020). Sleep quality during the COVID-19 pandemic: Not one size fits all. Sleep Med..

[38] Salfi F., Lauriola M., D’Atri A., Amicucci G., Viselli L., Tempesta D., Ferrara M. (2021). Demographic, psychological, chronobiological, and work-related predictors of sleep disturbances during the COVID-19 lockdown in Italy. Sci. Rep..

[39] Chen H.C., Lin C.M., Lee M.B., Chou P. (2011). The relationship between pre-sleep arousal and spontaneous arousals from sleep in subjects referred for diagnostic polysomnograms. J. Chin. Med. Assoc..

[40] Roth T. (2007). Insomnia: Definition, prevalence, etiology, and consequences. J. Clin. Sleep Med..

