# Supplementary material for: Insomnia, Pre-Sleep Arousal, Psychosocial Factors and Changes in Sleep Pattern during the Second Wave Lockdown of the COVID-19 Pandemic in Georgia
Source: Brain Sci. 2021 Dec 24;12(1):17. doi: 10.3390/brainsci12010017 (PMC8773778; doi:10.3390/brainsci12010017)
Supplement: Supplementary file 1 [file brainsci-12-00017-s001.zip › brainsci-1504592-supplementary.pdf]

Table S1. Pearson's and Spearman's correlations between all the study variables

| **Variables** | **1.** | **2.** | **3.** | **4.** | **5.** | **6.** | **7.** | **8.** | **9.** | **10.** | **11.** | **12.** | **13.** | **14.** | **15.** | **16.** | **17.** |
|---|---|---|---|---|---|---|---|---|---|---|---|---|---|---|---|---|---|
| 1. Age | | −0.05 | −0.38** | −0.55** | −0.29** | −0.04 | 0.22** | −0.06 | −0.13** | −0.05 | −0.11** | −0.27** | −0.11** | −0.26** | −0.13** | −0.15** | −0.31** |
| 2. Sex | | | −0.03 | 0.04 | −0.03 | 0.04 | −0.02 | −0.07* | −0.01 | 0.01 | −0.04 | −0.09** | −0.17** | −0.14** | −0.12** | −0.09** | −0.08** |
| 3. Marital status | | | | 0.30** | 0.11** | 0.05 | −0.04 | −0.03 | 0.01 | −0.02 | 0.07* | 0.11** | 0.03 | 0.14** | 0.07* | 0.05 | 0.13** |
| 4. Education | | | | | 0.38** | 0.01 | −0.07* | 0.07* | 0.08** | 0.10** | 0.15** | 0.20** | 0.07* | 0.18** | 0.09** | 0.18** | 0.20** |
| 5. Employment | | | | | | 0.07* | −0.01 | 0.11** | 0.07* | 0.11** | 0.12** | 0.23** | 0.15** | 0.19** | 0.14** | 0.15** | 0.15** |
| 6. Economic status | | | | | | | 0.04 | 0.04 | 0.13** | 0.06 | 0.05 | 0.05 | 0.11** | 0.05 | 0.04 | 0.02 | 0.04 |
| 7. Chronic disease | | | | | | | | 0.04 | 0.04 | 0.05 | 0.04 | −0.04 | 0.01 | −0.01 | -0.02 | 0.08** | −0.03 |
| 8. COVID-19 infection | | | | | | | | | 0.06 | 0.04 | 0.10** | 0.08* | 0.06* | 0.06* | −0.01 | 0.16** | 0.10** |
| 9. Family environment | | | | | | | | | | 0.21** | 0.23** | 0.24** | 0.26** | 0.30** | 0.19** | 0.22** | 0.20** |
| 10. Access to medical services | | | | | | | | | | | 0.17** | 0.16** | 0.15** | 0.16** | 0.10** | 0.19** | 0.13** |
| 11. ISI | | | | | | | | | | | | 0.41** | 0.36** | 0.43** | 0.24** | 0.52** | 0.61** |
| 12. PSS-4 | | | | | | | | | | | | | 0.33** | 0.48** | 0.21** | 0.41** | 0.49** |
| 13. Anxiety | | | | | | | | | | | | | | 0.63** | 0.41** | 0.38** | 0.42** |

| | | | |
|---|---|---|---|
| 14. Depression | 0.43** | 0.42** | 0.47** |
| 15. Social isolation | | 0.19** | 0.29** |
| 16. PSAS-Somatic | | | 0.61** |
| 17. PSAS-Cognitive | | | |

*$p < 0.05$; ** $p < 0.01$
